# Intellectual disability, exercise and aging: the IDEA study: study protocol for a randomized controlled trial

**DOI:** 10.1186/s12889-020-09353-6

**Published:** 2020-08-20

**Authors:** Guillermo R. Oviedo, Casimiro Javierre, Manel Font-Farré, Nauris Tamulevicius, María Carbó-Carreté, Arturo Figueroa, Susana Pérez-Testor, Josep Cabedo-Sanromá, Sarah J. Moss, Núria Massó-Ortigosa, Myriam Guerra-Balic

**Affiliations:** 1 Faculty of Psychology, Education and Sport Science Blanquerna, University Ramon Llull, Barcelona, Spain; 2 School of Health Science Blanquerna, University Ramon Llull, Barcelona, Spain; 3 Department of Physiological Sciences, School of Medicine, University of Barcelona, Barcelona, Spain; 4 Department of Health Sciences and Human Performance, College of Natural and Health Sciences, The University of Tampa, Tampa, Florida, USA; 5 Faculty of Psychology, University of Barcelona, Barcelona, Spain; 6 Department of Kinesiology and Sport Management, Texas Tech University, Lubbock, TX USA; 7 Physical Activity, Sport and Recreation Research Focus Area, Faculty of Health Sciences, North-West University, Potchefstroom, South Africa

**Keywords:** Intellectual disability, Exercise, Aging, Health-related fitness, Arterial stiffness, Quality of life

## Abstract

**Background:**

People with intellectual disabilities (ID) have low levels of physical activity (PA) together with accelerated aging profiles. Adherence to PA interventions for persons with ID is low based on barriers such as motivation. The IDEA study aims to determine the effect of two types of exercise programs, continuous aerobic (CAEP) vs sprint interval training (SIT), designed for seniors with ID on health-related physical fitness, cardiovascular parameters, quality of life (QoL), and emotional and cognitive function.

**Methods:**

In this trial, ninety seniors with ID between the ages of 40 and 75 yrs. from occupational health centers from the Autonomous Region of Catalonia (Spain) will be recruited. Participants will be randomly allocated to the CAEP, SIT, and control group. Both intervention groups will train 3 days/week, 1.5 h/day over 6 months. Outcome variables will be assessed at baseline, 6 months and 12 months. The outcome variables include weight, height, body composition, cardiorespiratory fitness, muscle strength, balance, flexibility, cardiovascular parameters (blood pressure, pulse-wave velocity, pulse-wave analysis), QoL and cognitive function. The intervention effect will be determined with mixed models with repeated measures to assess changes in the outcome variables over time (baseline to month 12) and between study arms. Relationship between variables will be analyzed with appropriate regression analyses.

**Discussion:**

Various studies reported on CAEP and SIT as exercise interventions for persons with ID with beneficial outcomes on body composition, fitness and blood pressure. To our knowledge, this is the first trial designed to analyse the positive changes on fitness, PA levels, cardiovascular, QoL and cognitive function promoted by CAEP training and SIT in seniors with ID. The findings of this study will assist in the development of more effective exercise interventions to ensure better compliance and adherence to exercise in seniors with ID.

**Trial registration:**

The trial is registered at the ISRCTN registry. Registration number: ISRCTN43594228. Registered 11 February 2019 – Retrospectively registered.

## Background

Persons with an intellectual disability (ID) present with alarming levels of sedentary lifestyles or very low levels of physical activity (PA) [1–3]. In addition, they present with greater multi-morbidity than persons without ID [4].

Although a direct relationship between health problems and low levels of PA or sedentary lifestyle has been established [5, 6], a large number of people do not meet the minimum levels of PA necessary for health benefits, especially older adults [7].

This relationship is of more concern in people with ID. The lack of support as well as multiple barriers faced by persons with ID to access sport facilities or exercise programs [8–10] put them in a vulnerable situation and at higher risk for suffering chronic diseases associated to sedentarism.

The aging process in people with ID starts around 40–50 years of age [11, 12]. Aging in adults with ID is associated with low physical fitness and health problems such as osteoporosis, diabetes, musculoskeletal disorders, dementia, hypertension and peripheral arterial disease [13, 14]. In Spain, adults with ID between 40 and 45 years old are considered older adults because the frequency of suffering these illnesses is similar to that of adults older than 65 years without ID [15].

Improved medical, healthcare and more person-centered care have gradually increased the mean life-expectancy of persons with ID [16]. Therefore, alternative and innovative solutions to maintain physical function and improve health and quality of life (QoL) of people with ID should include PA and exercise training.

To achieve clinically significant health-benefits and impact on health-related physical fitness, it is important to progress exercise volume and intensity [17]. Further, to maintain functional independence, cardiovascular health, muscle strength and prevent falls, resistance training should be included in multi-component exercise programs for people with ID [17–19]. Exercise programs for people with ID should take into account these general recommendations, without forgetting that delivering PA programs tailored to the specific needs of adults with ID is challenging because they need specific support, additional encouragement, and often, they may stop exercising if any discomfort is perceived [20].

Several exercise interventions for adolescents, young adults, middle-aged adults and seniors with ID implemented continuous aerobic exercise programs (CAEP), which is characterized by prolonged bouts of exercise at continuous steady-state intensity. Also, these interventions used CAEP combined with resistance, balance and flexibility [21–25]. They reported beneficial effects on peak oxygen consumption (VO_2_ peak), strength, balance and flexibility. Moreover, one study reported improvements in motor coordination and blood pressure [26]. In young adults, middle-age adults and seniors without ID, CAEP showed positive effects on improving brachial artery responses and central arterial stiffness [27, 28].

On the other hand, sprint interval training (SIT) is characterized by efforts performed at intensities ≥100% of the aerobic power and includes all-out or supramaximal short efforts (5–30-s) interspersed with active or passive recovery periods [29, 30]. Two studies conducted SIT programs in adolescents and young adults with ID [31] and adults with Down syndrome [32]. The authors reported improvements on VO_2_ peak, muscle endurance and blood pressure. In the general population, this exercise modality has been proposed as a time-efficient alternative strategy. Research studies reported that SIT elicited similar improvements in peripheral vascular structure and function [33], arterial stiffness [34], and VO_2_ peak than CAEP [35, 36]. Short bouts of all-out exercise may be an interesting time-efficient and more effective alternative to implement for training in seniors with ID who lack motivation for continuous exercises.

To the best of our knowledge, there is a lack of evidence in the literature documenting whether multi-component continuous or SIT exercise programs designed for seniors with ID will promote more significant benefits on health-related physical fitness, cardiovascular parameters, QoL, and emotional and cognitive function.

## Methods

### Study aims

The present trial aims to evaluate and compare the effectiveness of two different 6-months exercise programs on the health-related fitness of seniors with ID. Secondary to the main objective, this study will compare the effects of these interventions on the blood pressure, arterial stiffness, pulse wave velocity, PA levels and sedentary behaviors of the participants. Lastly, we seek to investigate the effect of the exercise programs on the QoL, emotional and cognitive function of the volunteers.

### Study design

The present study protocol describes a longitudinal three-armed parallel-group randomized controlled trial to evaluate and compare the effectiveness of two different multi-component exercise programs for seniors with ID.

A total of 90 participants from occupational day centers from Catalonia (Spain) will be recruited and randomly allocated to the CAEP, or SIT program or control group (CG). Participants from both exercise groups will receive a 24-week supervised exercise program, 3 times/week, 90 min/session. Participants from the CG will be visited by the researchers once a week to observe if they maintain their usual lifestyle. Outcome variables will be assessed at baseline (W − 1), month 6 (W 24; end of intervention) and month 12 (W 48; 6 months follow-up). After month 12, the CG will receive the SIT intervention in order to experience similar exercise-related health-benefits.

**Fig. 1:**
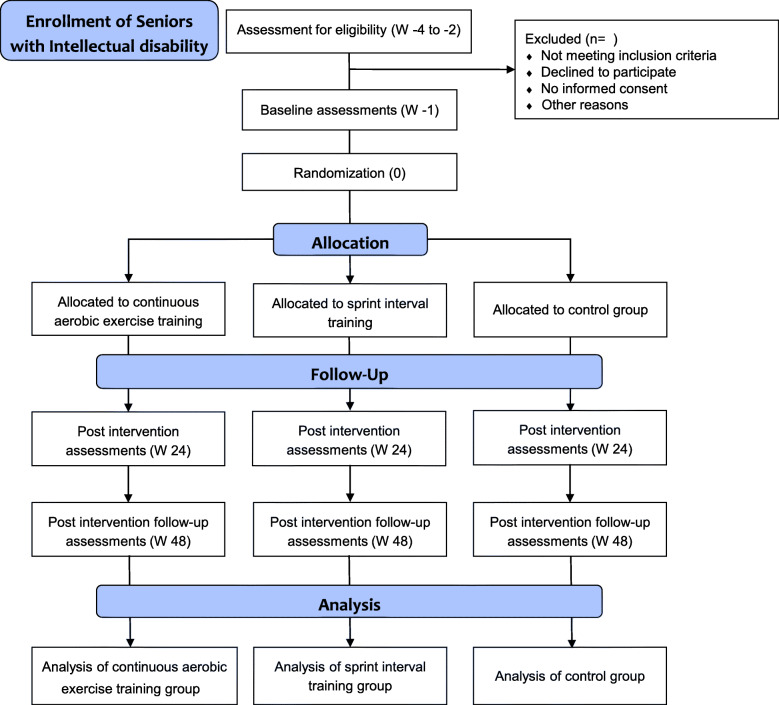
Proposed participant flow chart (CONSORT diagram). Abbreviation: W = week

This study was approved by the Institutional Review Board (CER URL 2017_2018_008) and complies with the principles of the Declaration of Helsinki [37]. The trial is registered as a current randomized controlled trial (registration number: ISRCTN43594228; registration date: 11 February 2019). Figure 1 presents the participant flow for this trial. The present protocol follows the Standard Protocol Items Recommendations for Interventional Trials (SPIRIT) guidelines and fulfills the SPIRIT checklist.

### Setting and recruitment

Occupational day centers for adults and seniors with ID from the Autonomous Region of Catalonia (Spain) will be contacted to be part of the present study. For those centers interested in getting a more detailed description regarding the project, the research team will inform the relevant professionals at each center about the background and aims of the study.

Interested adults between the ages of 40 and 75 yrs. diagnosed with mild to moderate ID from the centers that volunteer to be part of the study, will be invited to the first meeting with their families/legal guardians and care-givers from the centers. During this meeting, the procedures of the trial, testing procedures, benefits, risks and the time required for the study will be explained. In addition, an information sheet about the research and an informed consent form for participants and parent(s)/legal guardian(s) will be distributed.

During the second visit and after the informed consent is signed, participants will be requested to bring a history of medical conditions. Participants will undergo a physical examination and complete a health screening questionnaire to disclose any pathology, contraindication and/or conditions that will preclude them from participating in exercise. Additionally, participants will complete a QoL questionnaire and the emotional and cognitive function assessments.

During the third visit, all volunteers will participate in familiarization sessions before baseline testing at the laboratory. The number of familiarization sessions will be repeated until the researcher observes that the participants are confident and able to perform all assessments correctly. Finally, during the fourth visit participants will complete the remaining assessments at the laboratory facilities. The same tests will be repeated at month 6 (end of training period) and month 12 (6 months follow-up).

Volunteers included in the present study and their parent(s)/legal guardian(s) will receive written feedback explaining the results and changes of the outcome variables at the end of the training period and at the end of the follow-up period. The participants will also be given a Polar M200 heart rate (HR) monitor (Polar Electro OY, Finland) to wear during the course of the study.

### Sample size

An average increase in VO_2_ peak of 0.2 L/min was reported in previous studies that implemented multi-component exercise programs for adults with ID [26, 32]. In the present study, a target increase of 0.2 L/min and a standard deviation of 0.1 L/min in VO_2_ peak has been used for sample size calculation [38]. Assuming a 95% confidence level, 5% alpha error, and 20% beta error, with a 1:1:1 ratio between groups, we will seek to recruit 24 participants per group. To allow for a drop-out rate of 20% throughout the study, a total of 90 participants, 30 participants in each group, will be required.

### Participants

To be part of this study, all participants should fulfill the following inclusion criteria: a) ≥ 40-year-old and ≤ 75-year-old; b) diagnosed with mild to moderate ID; c) able to participate in activities in groups of 10 people; d) able to follow an exercise program on cycle ergometer; e) able to wear an accelerometer; f) able to walk independently; g) able to understand and perform all the fitness assessments; h) have medical clearance to perform exercise; and i) the participant and his/her parent(s)/legal guardian(s) should be willing to provide written consent.

Exclusion criteria will be: a) participants diagnosed with severe or profound ID; b) atlantoaxial instability; c) inability to walk unaided and unassisted; d) contraindications to exercise; e) use of medications that may influence the participants’ response to exercise; f) inability to communicate in Spanish/Catalan; g) unable to provide written informed consent; and h) parent(s)/legal guardian(s) not willing to provide written informed consent.

### Informed consent

A rigorous process and procedure of obtaining consent for the study will be set out to allow participants to be fully informed, autonomous, and empowered to consent. Participants and parent(s)/legal guardian(s) interested in being part of the study will review the documents with a member of the research team, allowing participants to ask questions or voice concerns.

Participants will be provided with adequate time to decide on participation in the study. To be part of the study, participants and parent(s)/legal guardian(s) must sign the informed consent form.

### Randomization and blinding

After baseline assessments and obtaining participants’ and parents’/legal guardians’ written informed consents, all volunteers will be centrally randomized to one of the three study arms (the CAEP, or SIT or CG) by using a stratified random-block randomization scheme. Three factors will arrange stratification: level of ID (mild or moderate), down syndrome (yes or not) and sex (male and female). Each of the 90 participants will be assigned with a unique serial number code generated by the Statistical Package for Social Science (SPSS) v 25.0 (IBM SPSS, Chicago, IL, USA) and randomly assigned in a 1:1:1 ratio to one of the three groups (*n* = 30 each). The serial number codes will be inserted into opaque envelopes that will be opened in the presence of the participant and parent(s)/legal guardian(s).

Because of the nature of the intervention, it is not possible blinding neither participants nor the exercise scientist delivering the interventions. Researchers in charge of the outcomes assessments and statistical analysis will be unaware of the participants’ allocations.

### Study intervention

The multi-component exercise programs will be delivered at the occupational day centers for adults with ID. All sessions are designed by experienced sports scientists and physiotherapists, following the American College of Sports Medicine (ACSM) and the National Strength and Conditioning Association (NSCA) guidelines [17, 39]. The different trainers in charge of delivering the interventions will undergo standardized training to ensure that the sessions offered at the various centers will be comparable. The multi-component exercise programs will include aerobic, resistance, balance and flexibility training.

Participants from the CAEP and SIT program will undergo a 24-week exercise program consisting of three sessions per week (72 exercise sessions), 90-min per session. Both groups will be divided into 3 groups; 10 participants/group and the participant-trainer ratio of 10:2. The programs will be divided within 3 phases: initial phase (week 1 to 6); improvement phase (week 7 to 16); and maintenance phase (week 17 to 24). Sessions for both groups will be matched for the total duration.

All sessions will include 10-min for standardized warm-up and cool-down exercises. Warm-up will be implemented to gradually increase the HR, body temperature, joint mobility and prepare the body of the participants for the exercises of the conditioning phase. After the conditioning phase, cool-down exercise will be performed followed by stretching exercise.

The resistance training will include a total of 22 exercises (see Additional file 1): 8 for upper-limbs (biceps curl, triceps extension, lateral shoulder raise, rotator cuff exercise); 8 for the torso (4 for lower and upper back: seated back extension, low row; reverse flies; standing high row; 2 for abdominals: side bend, seated crunch; and 2 for chest: wall push-off, standing chest press); and 6 for lower limbs (squats, hip abduction/adduction, knee extension/flexion, and calf raise).

All the exercises will be performed with yellow and red elastic bands (Thera-Band®, The hygienic Corporation, Akron, OH) or participants’ body weight. To control the intensity of the strength training, we will use the 0 to 10 points RPE OMNI-RES scale [40] designed for resistance training with elastic bands.

The intensity of the resistance training will increase during each phase (Table 1).

**Table 1:** Intensity of the resistance training

Phase	Sets	Repetitions	Rest between sets	Intensity (RPE)
1: Initial	3	12–15	1′ to 2’	5–6
2: Improvement	3	10–15	1′ to 2’	7
3: Maintenance	3	12–15	1′ to 2’	8

Balance training is designed to challenge central and peripheral mechanisms involved in the control of movement and stability (vestibular; visual and somatosensory). The training will start with static positions on the floor, progressing from double-leg stance with feet apart and together, to semi tandem and tandem stance, and one-leg stance. Subsequently, the participants will perform the same exercises with eyes closed.

Once the participants can hold these positions (10–30 s), we will introduce balance pads and will follow the above-explained progression.

During the subsequent cycles, participants will perform progressively challenging dynamic activities which will consist of toe-to-heel walk, walking on a line, side walking, reverse walking, zig–zag walking, longer strides, tandem walking and obstacle courses with cones. These activities will be performed on different surfaces and at different elevations.

Finally, increasingly challenging dual tasks activities such as turning their head toward a visual target during walking will be introduced.

Flexibility exercises will be executed after the cool-down period of each session. We will use sustained stretches for each major muscle group targeted during the session. Active and passive static stretching exercises will be used, holding the position for 10–30-s at a point of tightness or slight discomfort [17].

Volume and intensities for warm-up, cool-down, resistance, balance and flexibility exercises will be similar for both intervention groups. The difference between the CAEP and SIT program consist in the cycling training modality.

Based on previous studies [41, 42], a training attendance ≥75% will be set as the minimum attendance rate to achieve the results expected. We will divide the participants between those who adhere and do not adhere to ≥75% of training sessions. Compliance and adherence with the exercise programs will be determined by the number of sessions attended and completed in accordance with the exercise protocols.

#### Continuous aerobic exercise program

**Fig. 2:**
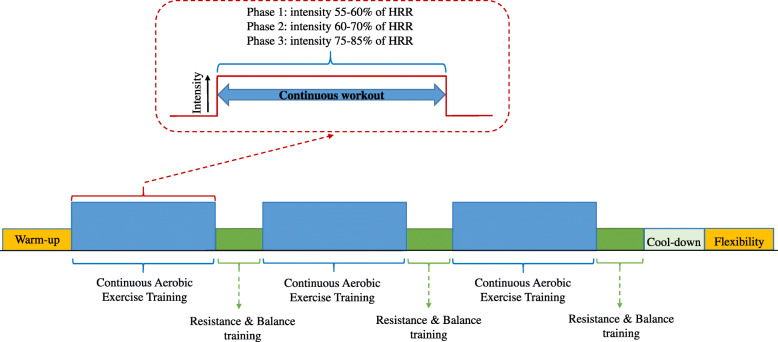
Sample of continuous aerobic exercise training intervention per session

As mentioned before, the CAEP will be based on a combination of aerobic, resistance, balance and flexibility exercises (Fig. 2). Aerobic training will be performed on cycle ergometers (Concept 2 BikeErg, Concept2 Inc., Morrisville, VT). During the program, the exercise on the cycle ergometer will start with 5-min of warm-up followed by three bouts of continuous cycling at a steady-state intensity. The length of the bouts (5 to 10-min) and intensity (55 to 85% of the HR reserve) will be progressively increased across each phase (Table 2). To ensure that the participants are exercising at the appropriate intensity, Polar M200 HR monitors (Polar Electro OY, Finland) will be worn.

**Table 2:** Intensity and duration of the continuous aerobic exercise program

Phase	No. of bouts per session	Bout duration (min)	Intensity (HRR)
1: Initial			
Week 1 to 3	3	5	55%
Week 4 to 6	3	7	60%
2: Improvement			
Week 7 to 9	3	8	60%
Week 10 to 12	3	9	65%
Week 13 to 16	3	10	70%
3: Maintenance			
Week 17 to 20	3	10	75%
Week 21 to 24	3	10	75–85%

#### Sprint interval training program

**Fig. 3:**
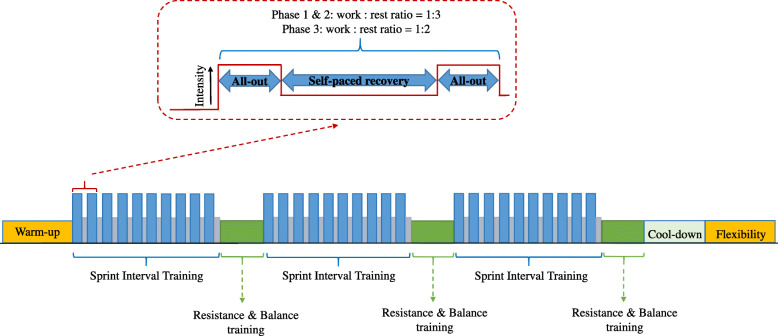
Sample of sprint interval training intervention per session

The SIT program will consist on a combination of aerobic, resistance, balance and flexibility exercises (Fig. 3). The SIT will be performed on cycle ergometers (Concept 2 BikeErg, Concept2 Inc., Morrisville, VT). During the SIT program, exercise will start with 5-min of warm-up followed by all-out SIT bouts. The SIT group will perform bouts of 5 to 10-min of exercise consisting of 5 to 20-s all-out sprints followed by 15 to 60-s of low cadence recovery (1:3 and 1:2 work-rest ratio). The duration of the sprints and the active recovery will be modified throughout the program (Table 3). The HR of the participants will be controlled by using Polar M200 HR monitors (Polar Electro OY, Finland).

**Table 3:** Intensity and duration of the sprint interval training program

Phase	No. of bouts per session	Bout duration (min)	No. of repetitions	Work: rest ratio
1: Initial				
Week 1 to 3	3	5	15	5″ – 15”
Week 4 to 6	3	7	21	5″ – 15”
2: Improvement				
Week 7 to 9	3	8	12	10″ – 30”
Week 10 to 12	3	9	9	15″ – 45”
Week 13 to 16	3	10	8	20″ – 60”
3: Maintenance				
Week 17 to 24	3	10	10	20″ – 40”

#### Control group

The participants of the CG will continue with their activities of daily living while receiving the usual care from their centers, which exclude exercise. Participants from the CG will receive Polar M200 HR monitors (Polar Electro OY, Finland) that will be used to control changes on their activities of daily living.

They will be visited be the researchers 2 to 3 times a week, during the 6 months of the intervention program. During these visits, the researchers will interview the participants to verify that their daily routines have not been modified. Given the benefits of exercise training and for ethical reasons, participants who completed the 12 months in the CG will receive SIT program for 6 months.

### Outcome measurements

**Fig. 4:**
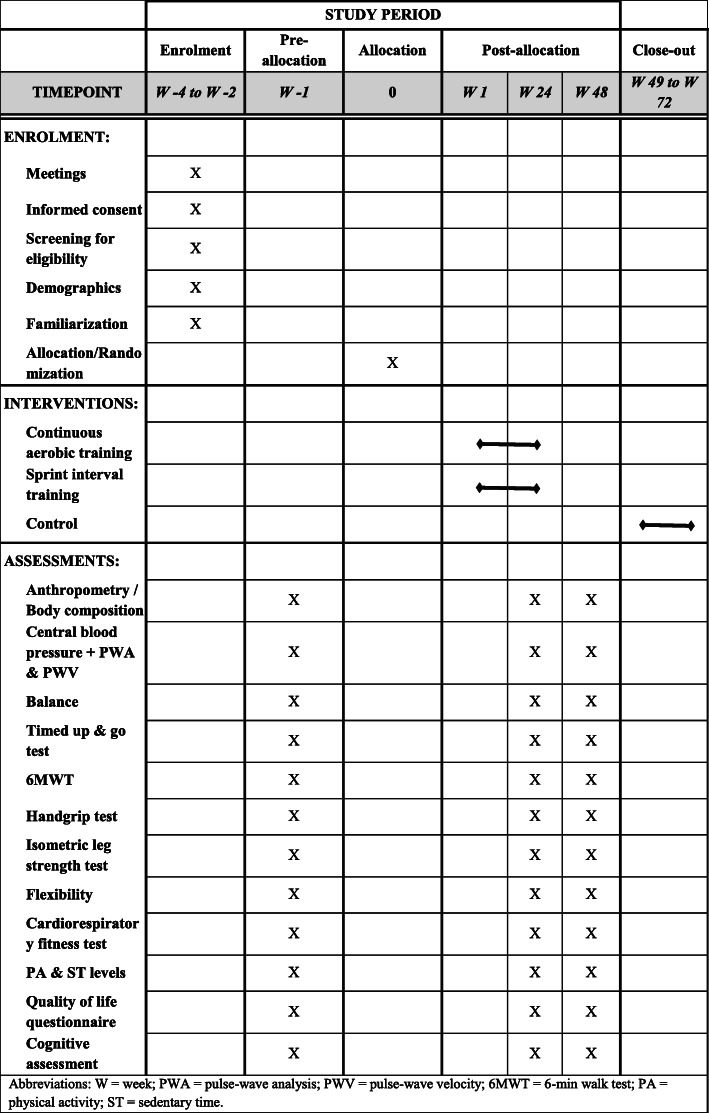
SPIRIT figure showing schedule of interventions, enrolment and assessments

Assessments will be conducted on three occasions over 12 months: at baseline = W − 1; at the end of the intervention = W 24 (6 months after baseline) and follow-up = W 48 (6 months after the end of the intervention) (Fig. 4 shows the SPIRIT Figure).

During baseline assessments, personal information regarding age; sex; ID level; ID etiology; living arrangement; education; medication, smoking and alcohol habits; gross annual household income will be obtained.

All tests will be conducted during the morning at a room temperature of 22–24 °C and relative physical humidity between 55 and 65%. Participants will be fasted for at least 3 h before the scheduled visit. Additionally, participants will be requested neither consume alcohol, caffeine (coffee, soda, tea, chocolate, etc.) or vasoactive medication for 12 h before testing visit nor engage in exercise for at least 24 h before the visit to the laboratory.

#### Primary outcome measurement

##### Maximal aerobic capacity

To assess the maximal aerobic capacity, the participants will perform a cardiopulmonary exercise test on a precalibrated cycle ergometer (Excalibur, Lode, Groningen, the Netherlands), cycling at 50–60 rpm. After a 1-min period cycling at 0 W, participants will follow a 10 W/min ramp protocol until exhaustion. Peak effort of the test will be considered when the participant achieves a plateau in HR or a respiratory exchange ratio (RER) ≥ 1.1 or at volitional exhaustion.

VO_2_ peak (L/min), relativeVO_2_ peak (ml/kg/min), minute ventilation (VE) (L/min) and RER will be measured breath-by-breath with an automatic gas analysis system (Metasys TR-plus, Brainware SA, La Valette, France) equipped with a pneumotachometer and making use of a two-way mask (Hans Rudolph, Kansas, USA). Gas and volume calibrations will be performed before each test, according to the manufacturer’s guidelines. Peak values will be recorded as the highest value during the last 30-s of exercise. Participants will be monitored continuously via a 12-lead electrocardiogram (CardioScan v.4.0, DM Software, Stateline, Nevada, USA).

#### Secondary outcomes measurement

##### Anthropometric measurements

Height will be measured to the nearest 0.1 cm with a stadiometer (Seca 225, Seca, Hamburg, Germany). Weight will be measured to the nearest 0.1 kg on a digital scale (Tanita MC-780 U, Arlington Heights, IL, USA), with the participant wearing lightweight clothing and no shoes. Body mass index (BMI) will be calculated as weight in kilograms divided by height in meters squared (kg/m^2^). Body fat percentage and fat-free mass percentage will be obtained via segmental (arms; trunk and legs) bioimpedance analysis (Tanita MC-780 U, Arlington Heights, IL, USA).

With a tape measure, waist circumference will be measured three times at the midway between the lower rib and the iliac crest on the midaxillary line. Hip circumference will be measured three times at the level of greatest protuberance of the buttocks.

##### Blood pressure, pulse wave and pulse wave velocity assessments

The brachial blood pressure will be measured with an automatic sphygmomanometer system (SphygmoCor Xcel, AtCor Medical, Sydney, Australia). A conventional brachial cuff will be inflated around the upper arm to measure brachial systolic and diastolic blood pressures which are calculated using the oscillometric technique. Brachial blood pressure and pulse waves are used to derive central aortic hemodynamic measures by applying proprietary digital signal processing and a validated mathematical transfer function [43–45]. Finally, we will obtain aortic blood pressure, the augmentation index (AIx), augmented pressure, forward wave pressure, and backward wave pressure [44, 46].

Central arterial stiffness will be determined by calculating the carotid-femoral pulse wave velocity (PWV), which is a well-established marker of aortic stiffness [47]. To calculate the PWV, applanation tonometry technique will be used to obtain carotid pulse waves, while simultaneously femoral pulse waves will be obtained from the femoral artery by a cuff placed around the upper thigh. PWV will be calculated using the ratio between the corrected carotid to femoral distance and the foot-to-foot transit time of the pressure wave from the carotid to the femoral sites. Carotid to femoral artery distance will be measured from (A) the carotid site of measurement to the suprasternal notch; (B) from the suprasternal notch to the proximal edge of the thigh cuff; and (C) from the femoral artery below the inguinal ligament to the proximal edge of the thigh cuff. Distances A and C will be subtracted from distance B and used in the calculation of PWV [43].

##### Balance assessments

The static balance will be assessed with a pressure platform (Podoprint Balance Platform, Namrol, Barcelona, Spain). All participants will perform a double leg stance with eyes open and closed. Participants will be instructed to stand erect on the platform without shoes, motionless and with their arms by their sides. Heels will be separated by 3 cm and toes forming a 30° angle. The software requires each participant to maintain this position for 52-s [26]. Three trials (3 trials with open eyes and 3 trials with closed eyes) will be performed. The total travel distance, radial area, mean mediolateral and mean anteroposterior displacements of the center of pressure will be measured at a frequency of 100 Hz using manufacturer’s specific software (PodoPrint v2.6, Namrol, Barcelona, Spain).

Static balance on a single leg will be assessed by the single leg stand test. Participants will be requested to stand without shoes and with their hands on the hips on a single leg, with the non-supporting foot placed against the inside knee. The time performing the static balance with a maximum of 30-s will be recorded. Three trials will be performed on each leg, and the best trial will be recorded. The reliability of this test was assessed in a previous study [48].

##### Functional tests

The timed up and go test will be used to assess the dynamic balance and gait speed. The test consists of rising from an armless chair with a seat height of ≈46 cm without using the arms, walking 9-mts away from the chair and going back to the chair sitting again. The time to complete the test will be recorded. The best trial out of three opportunities will be used in data analyses. Reliability of this test in people with ID was previously assessed by Blomqvist et al. (2012).

The 6-min walking test (6MWT) will be used to assess mobility and submaximal exercise performance. Participants will walk at a self-paced velocity for 6-min in a 30 m unobstructed indoor corridor. They will be asked to walk up and down, without running or jogging, as fast as they can and as far as possible within 6-min. The covered distance will be measured to the closest 0.5 m. Reliability and validity of the test were obtained for adults and seniors with ID in a previous study [49].

##### Hand-grip isometric test

Maximal isometric handgrip strength will be assessed by using a digital hand-grip dynamometer (TKK 5401, Takei Scientific Instrument Co., Ltd., Tokyo, Japan).

Participants will be instructed to seat comfortably on an armless chair. The participant will be seated with the trunk upright, elbow flexed at 90°, forearm and wrist in a neutral position and not touching the trunk. Three maximal voluntary contractions of 3–5-s will be performed with both the left and right hand, alternating hands and ensuring a recovery of 1-min between repetitions to avoid muscle fatigue. The best of the three trials of each extremity will be recorded to the nearest 0.1 kg.

After a further 3-min recovery period, we will assess the ability to maintain the maximal isometric hand-grip strength. Seated on an armless chair, participants will be asked to maintain an isometric hand-grip contraction for 10-s with each hand. Peak, maximum and average isometric strengths will be recorded to the nearest 0.1 kg with an electronic hand-grip dynamometer (Powerlab4/20THandgrip, ADInstruments, Bella Vista NSW, Australia).

##### Lower limbs isometric strength assessment

**Fig. 5:**
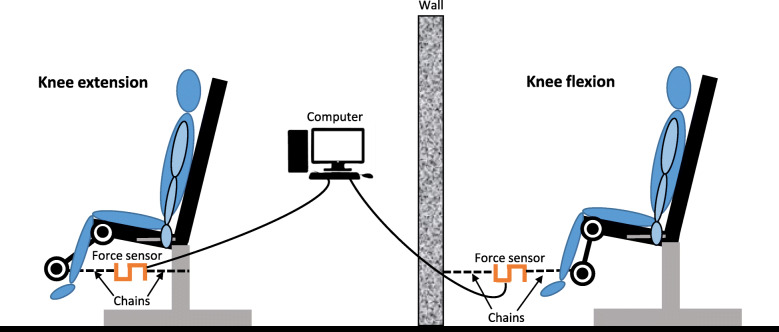
Assessment of the maximal isometric knee extension and flexion

Maximal isometric strength of knee extensor and flexor muscles will be assessed with the participant seated on a knee flexion/extension machine (Salter® M-126, Barcelona, Spain) with hips and knees flexed at 90°. A strength sensor, connected to a computer with specific software for data analyses (Chronojump v.1.9.0, Barcelona, Spain), will be attached to the knee flexion/extension machine to test lower limbs isometric strength of each participant (Fig. 5). The test will consist of 3 maximal voluntary contractions (3 for extension and 3 for flexion) of 3–5-s each with 1-min rest between contractions. First, we will test the extensor muscles of the knee with the dominant leg, then the non-dominant leg and finally both legs simultaneously (total of 9 repetitions). Next, the same procedure will be followed for the knee flexor muscles. The best score of each trial will be recorded.

##### Lower limbs muscle endurance

Muscle endurance of the lower limbs will be assessed by using the 30-s chair stand test [50]. Participants will be asked to sit down and stand upright (full stance) as often as possible during 30-s without using their hands with arms folded across the chest. Three trials will be performed with a recovery of 3-min between trials. The maximum number of repetitions achieved during one trial will be recorded. The test presents good reliability in seniors (ICC = .98) [50].

##### Flexibility measurements

Flexibility will be assessed by using the chair sit-and-reach test (CSRT) and the back scratch test (BST) [50]. In the CSRT, which assess the flexibility of the hamstrings, the participant sits on a chair with one leg extended and the other leg flexed. The person is instructed to bend forward with the arms extended to touch the toes of the extended leg and maintain the position for 2–5 s. If participants do not reach the 0-point (end of the shoe), negative values will be recorded; otherwise, positive values will be recorded if they reach behind the 0-point. The best of three trials will be recorded to the nearest cm. The reliability of the test for older adults was good (ICC = .92 and .97) [51].

Shoulder flexibility will be assessed by the BST in the upright position. The extremity to be valued is above the shoulder on the same side, elbow pointing upward, the palm facing inwards and downwards and fingers extended. The other arm is placed behind the back, palm facing outward and fingers extended up the middle of the back. The distance (cm) between extended middle fingers of both hands will be assessed. The distance that is missing to join the extended middle fingers is classified as negative distance and the overlap of these, is a positive distance. Three trials will be performed by either side, recording the best of them. The reliability of the test for older adults is good (ICC = .97) [52].

##### Physical activity and sedentary behaviors assessment

To assess the PA and sedentary level of the volunteers we will use GT3X ActiGraph accelerometers (ActiGraphTM, Fort Walton Beach, FL, USA; Firmware 1.9.2) and data will be downloaded with the ActiLife 6 Software v.6.13.4.

Participants and parent(s)/legal guardian(s) will receive face-to-face instructions on how to wear the accelerometer (correct positioning and orientation) during all waking hours, as well as its placement and wear time. We will reinforce these instructions by giving an information sheet to each participant and parent(s)/legal guardian(s), along with pictures showing correct positioning of the device. The accelerometers will be fitted to the participants’ right hip and will be collected after eight days. The devices will not be worn during water-based activities.

Outcome variables will be total PA (counts/min), steps per day (steps/day), and time spent (min/day) in sedentary (0 to 99 cpm), light PA (100 to 2019 cpm) and moderate-to-vigorous PA (≥ 2020 cpm). We will replicate the intensity cutoff points from the National Health and Nutritional Examination Survey (NHANES) for adults over 18 yrs. [53]. Non-wearing time will be defined as a period of 60 consecutive minutes of zero counts with an allowance for up to 2-min of counts between 1 and 100 [54].

To have valid data, participants will have to wear the accelerometer during waking hours for ≥10 h/day [53, 55] for ≥4 (week and/or weekend) days [56, 57].

##### Quality of life assessment

Quality of life will be assessed by using the Spanish version of the Personal Outcomes Scale (POS) [58, 59]. The validity and reliability of this test in Spanish people with ID was assessed by Carbó-Carreté et al. (2015).

Personal outcomes are generally defined as valued personal experiences and circumstances that follow as a result of some activity, intervention or service. The POS measures the QoL outcomes based on eight dimensions which are arranged into three higher-order factors [60, 61]: *independence*, which encompasses personal development and self-determination; *social participation*, composed of interpersonal relations, social inclusion, and rights; and *well-being*, which includes emotional well-being, physical well-being, and material well-being. The POS aims to assess QoL in people with ID based in the above-mentioned domains and is divided into three information sources:

- Self-report: assessment of subjective perspective of QoL with individual participants answering on his/her own.
- Report by the professional: assessment of the individual’s experiences and circumstances from the view of direct care staff or a service technician.
- Family report: the indicators are scored by a family member’s perspective.

Each of the eight core QoL domains has 6 items, which means that a total of 48 items are answered, using the following calculation: (3) = always, (2) = sometimes, and (1) = rarely or never. Scoring ranges from 48 to 144 for each information source, with higher scores representing better QoL.

##### Emotional and cognitive function assessments

Emotional and cognitive assessment will be performed by using the Human Figure Drawing Test (HFD test) [62]. This is a graphic non-verbal projective test that allows knowing emotions, body self-perception of body scheme, self-esteem, anxiety and aggressiveness, between others [62–64].

The HFD test has been applied in persons with ID, including Down syndrome, and other populations, and has been considered a useful instrument [63, 65–67].

After giving a blank DIN-4 paper and HB2 pencil to the participants while they are at the exercise intervention facility, the researcher will provide the following verbal instructions: “Draw a person, their full body, the best you can”. The participants will have a maximum of 15 min to perform the drawing and they will not be able to erase any part of the drawing to make corrections. The cognitive function analyses will be performed with the drawing from the HFD test by applying the Draw a Person Intellectual Ability Test for Children, Adolescents and Adults (DAP-IQ test) [68]. The DAP-IQ test evaluation system is based on 23 quantitative indicators of the drawing [68].

For the emotional analysis, we will use the Home-Tree-Person (HTP) test [69]. The HTP test implements a qualitative analysis of the characteristics, size and perspective of the drawing produced with the HFD test [69].

### Occurrence of adverse events

Participants may experience some unlikely risks or undesirable effects during the assessment of the outcome variables and PA programs. They may suffer some discomfort, fatigue or muscle soreness. These discomforts and muscle soreness are typical in sedentary people who start a PA program, but after 2 or 3 sessions these discomforts tend to disappear.

In the event that any participant suffers any discomfort, pain or problems during the evaluations or during the study, he/she will have the possibility to discuss about it with the Principal Investigator or the project coordinator the same day. In case the participant might need more counseling, an opportunity will be arranged to further discuss about the possible cause of discomfort with the Principal Investigator or the supervisor. If the participant agrees, the participant’s general practitioner will be informed so that he/she can follow up in case of injury or illness.

All adverse events as well as serious adverse events will be carefully monitored and we will record the day and time of the occurrence. The Principal Investigator and local IRB will be informed about any adverse event.

### Data management and monitoring

All data collected by the research team will be collected by means of the unique number assigned to each participant and entered at the data collection site. All data and personal information present on questionnaires and data sheets will be anonymize. In order to ensure the security of personal information and data, we will use security procedures such as encryption and password protection as a standard practice. Personal information and data collected will only be handled by the Principal Investigator and project coordinator, who will anonymize the data for data analyses by collaborators.

The Principal Investigator, the project coordinator, and an independent researcher who will not be involved in the trial will comprise the Data Monitoring Committee (DMC). On a monthly basis the DMC, which is independent from the sponsors and competing interests, will meet for data control and quality and ensure its validity. In case of aberrant outcomes and/or missing data, the DMC will contact the pertinent participant and researchers.

### Statistical analysis

Outcome analysis will be performed blinded to group allocation. The descriptive analysis of the sample will be expressed as mean and standard deviation for continuous and normally distributed variables, meanwhile non-normal distributed variables will be presented as median and interquartile range. Categorical variables will be presented as number and percentage with their standard error. Normality and homogeneity will be tested by using Kolmogorov-Smirnov test and Levene’s test, respectively. When necessary, appropriate transformations will be applied in case of non-normal distributions.

The analysis of the data set will follow an intention-to-treat design and all randomized participants for whom baseline assessments were conducted will be included in the final analysis.

To analyze the outcomes of the study we will use linear mixed-effect models with repeated measures. We will assess changes over time (W -1; W 24; W 48) and between study arms. Appropriate post-hoc tests will be implemented to analyze within and between group differences of the study arms at each time point. Categorical variables will be analyzed using Pearson’s χ-squared or Fisher’s exact test at baseline, after intervention and follow-up period.

To examine the correlations between the different outcomes, Pearson’s correlations and multiple regression analysis will be performed.

If necessary, baseline differences between the trial arms on outcomes variables will be used as covariates and fitted as factors in the models.

Two-sided tests of statistical significance will be used in all analyses. Effects size of the interventions will be presented in addition to significance tests. Significance levels will be set at an alpha level less than 5%. Statistical analyses will be conducted using SPSS v. 25 (IBM SPSS Statistics, Chicago, IL, USA).

## Discussion

To counteract the high prevalence of cardiovascular risk factors, decreased physical fitness [70–72], premature aging [11, 73], and overcome different barriers that seniors with ID have to face to live and active life [9, 74], it is essential to design exercise programs that best suit their needs. Therefore, the main objective of this study is to design two specific PA programs for seniors with ID and determine which of them will generate the greatest benefits in health-related physical fitness, cardiovascular parameters, QoL and emotional and cognitive aspects.

This study will span 12 months. After 6 months, participants will be followed up for 6 months without training. Finally, 12 months after starting the study, all participants in the CG will be offered the possibility to participate in the SIT program for 6 months.

Previously, numerous multi-component programs implemented continuous aerobic training in adolescents [22, 75], adults [24–26, 76, 77] and seniors with ID [21, 23, 78]. Also, some studies implemented SIT programs for adolescents and young adults with ID [31] and with Down syndrome [32]. Nevertheless, none of these studies implemented SIT programs specifically designed for seniors with ID. Furthermore, the present study will try to elucidate if exercise interventions promote beneficial changes on cardiovascular measures of central and peripheral arteries and which of these programs will have the most significant effect on the outcome variables.

Given the direct associations between cardiorespiratory fitness and mortality [79], muscular fitness and mortality [80], and the important effects of high intensity interval training programs on muscular adaptation and remodeling [81], it is necessary to further investigate the effects of SIT programs on overall fitness and vascular function in seniors with ID.

An increasing number of studies have reported positive effects of different exercise programs on vascular function and structure in adults without disabilities [82–84]. On the other hand, 12 weeks of CAEP reduced arterial stiffness in young adults with ID [85]. Nevertheless, modalities of exercise training such as short intermittent-bouts of high-intensity exercise, should be further explored in adults without intellectual disabilities and, even more, in adults and seniors with ID. In addition, the present study will help us to understand whether the mode of exercise training (CAEP vs SIT) plays a role in central and peripheral cardiovascular improvements. On top of that, this trial will complement available descriptive data on arterial stiffness and will give information regarding the changes on aortic stiffness and hemodynamics at mid-term (6 months) and whether these changes are maintained after the end of the programs (12 months).

In the present trial we will also evaluate qualitative parameters such as QoL and cognitive and emotional aspects of participants with ID. We consider that QoL in seniors with ID is a very important parameter, which is influenced by PA and sports practice [86, 87]. Beside the positive effects on QoL, PA may also improve cognitive and emotional aspects in people with ID, as previous studies have shown after implementing dance programs [64, 88].

It was reported that high-intensity exercise of short duration appears to produce lower effort perception values than continuous or longer duration exercise [89], and it seems that enjoyment of and preferences for interval exercise are equal or greater than for continuous exercise [90]. Moreover, shorter high-intensity protocols may have the potential to reduce some of the barriers to exercise and promote positive psychological responses [91, 92]. A study found that high-intensity interval exercise is as feasible as continuous moderate intensity exercise in adults with severe mental illness [93]. Thus, SIT programs may be a feasible and effective strategy to increase exercise adherence in seniors with ID.

The findings of this trial will help the development of new exercise interventions, prescription and strategies designed to promote physiological and psychological positive changes in seniors with ID. We believe that prescribing SIT programs aimed at reducing health problems, sedentarism and increasing PA levels is a meaningful research effort that is of great value.

Finally, it is extremely relevant to further understand the effects of SIT on the anticipated outcome variables, because if the results are positive, this could have important implications for the prescription and adherence to exercise in seniors with ID.

### Limitations

We would like to highlight some limitations of the IDEA study. First, we are not including seniors with severe/profound ID. This is due to the fact that these persons need one-to-one supports to perform their activities of daily living, which makes it impossible to cover the costs implications. Second, it is not possible to control all exercises and activities that the participants will perform outside the intervention programs and this may influence the results of the study. However, all participants will use activity monitors that will assist us to control for those activities conducted outside the study programs.

## Supplementary information

**Additional file 1.** Resistance exercises of the IDEA Study.

## Data Availability

Data sharing is not applicable to this article as no datasets were generated or analysed during the current study. Relevant data from this study will be made available upon study completion and researchers request from the corresponding author.

## Abbreviations

ID: Intellectual disability
PA: Physical activity
QoL: Quality of life
CAEP: Continuous aerobic exercise program
VO_2_ peak: Peak oxygen consumption
SIT: Sprint interval training
CG: Control group
W: Week
SPIRIT: Standard Protocol Items Recommendations for Interventional Trials
HR: Heart rate
SPSS: Statistical Package for Social Science
ACSM: American College of Sports Medicine
NSCA: National Strength and Conditioning Association
BMI: Body mass index
AIx: Augmentation index
PWV: Pulse wave velocity
6MWT: 6-min walking test
CSRT: Chair sit-and-reach test
BST: Back scratch test
RER: Respiratory exchange ratio
VE: Minute ventilation
NHANES: National Health and Nutritional Examination Survey
POS: Personal Outcomes Scale
HFD: Human Figure Drawing
DAP-IQ: Draw a Person Intellectual Ability Test for Children, Adolescents and Adults
HTP: Home-Tree-Person
DMC: Data Monitoring Committee
IRB: Institutional Review Board
