# Supplementary material for: Intellectual disability, exercise and aging: the IDEA study: study protocol for a randomized controlled trial
Source: BMC Public Health. 2020 Aug 20;20:1266. doi: 10.1186/s12889-020-09353-6 (PMC7439526; doi:10.1186/s12889-020-09353-6)
Supplement: Supplementary file 1 — Additional file 1. Resistance exercises of the IDEA Study. [file 12889_2020_9353_MOESM1_ESM.docx]

**Upper limbs exercises**


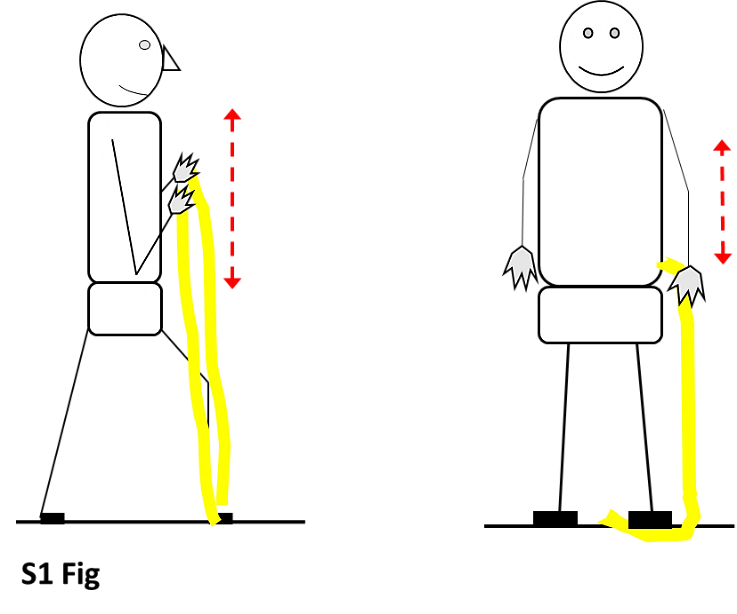


**Exercise 1 & 2.** Bilateral and unilateral biceps curl.


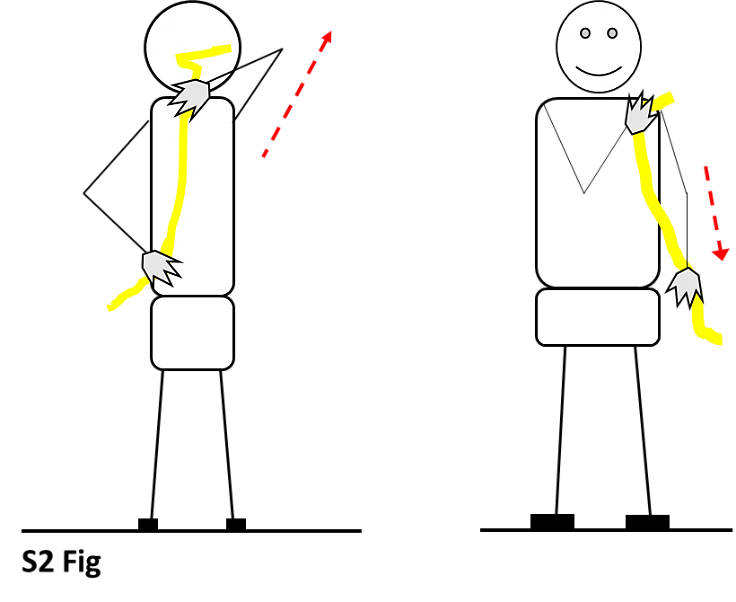


**Exercise 3 & 4.** Triceps extension.


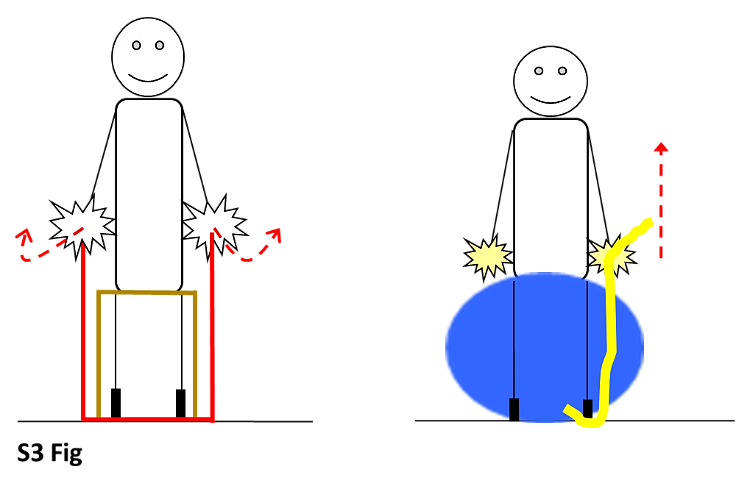


**Exercise 5 & 6.** Bilateral and unilateral shoulder raise.


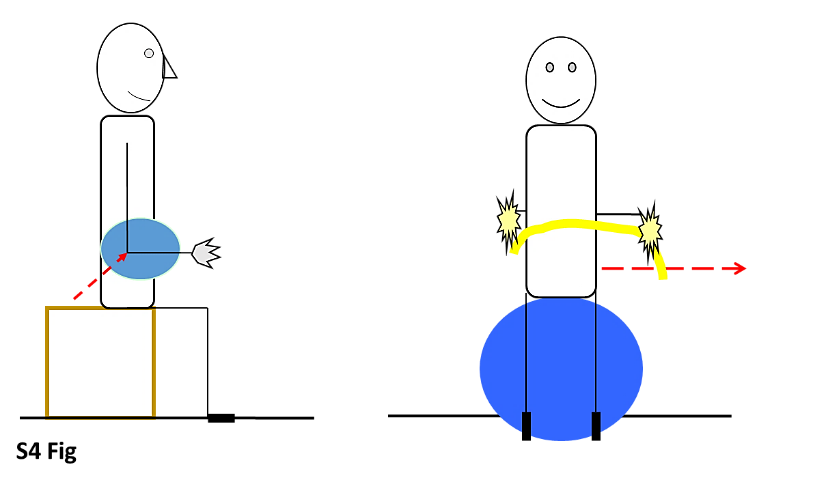


**Exercise 7 & 8.** Rotator cuff exercise (internal and external rotation).

**Exercises for the torso**


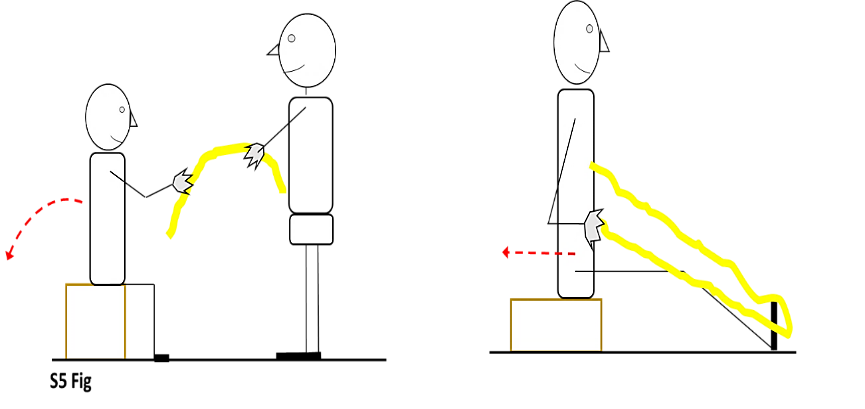


**Exercise 9 & 10.** Seated back extension and low row.


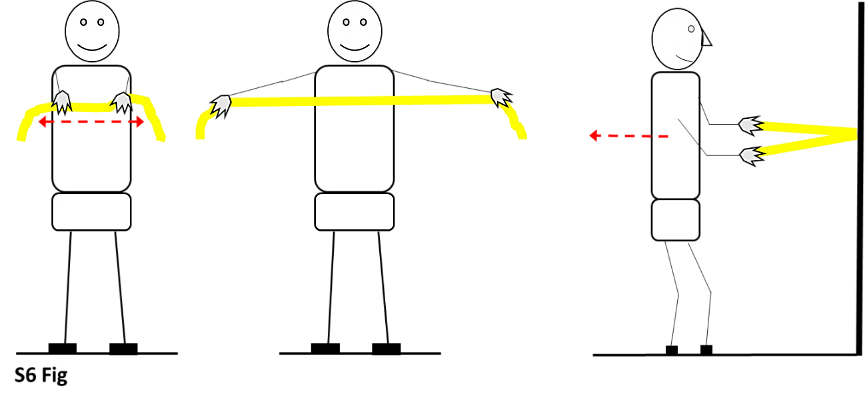


**Exercise 11 & 12.** Reverse flies and standing high row.


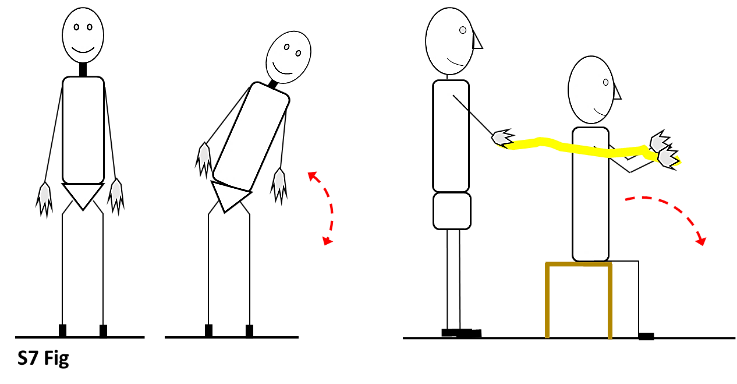


**Exercise 13 & 14.** Side bend and seated crunch.


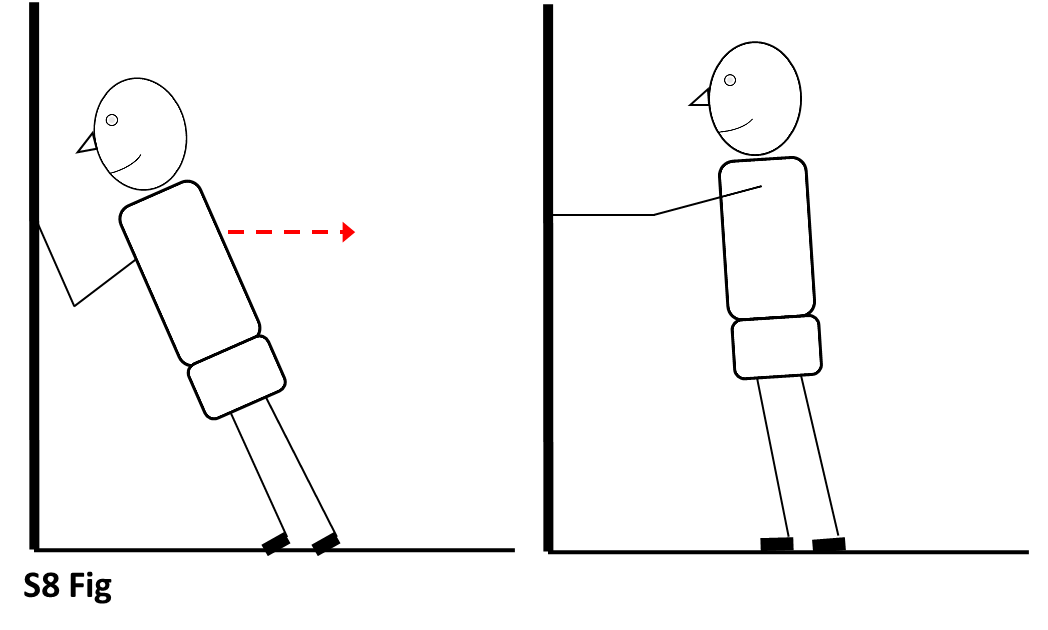


**Exercise 15.** Wall push-off.


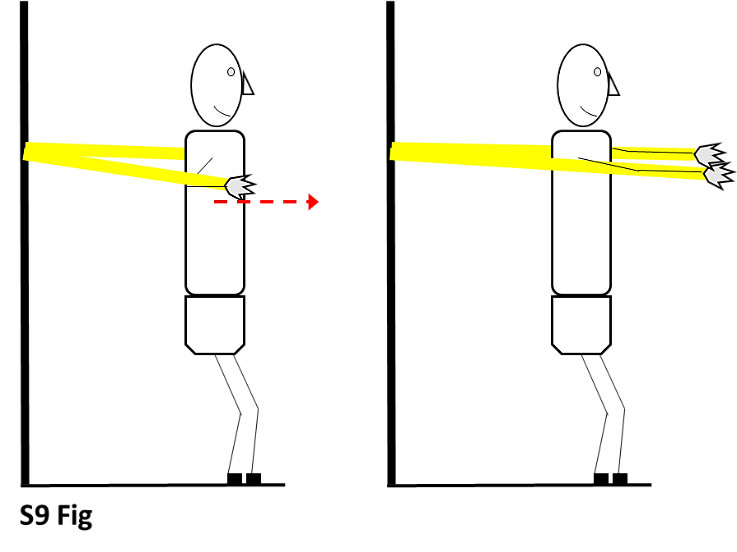


**Exercise 16.** Standing chest press.

**Lower limbs exercises**


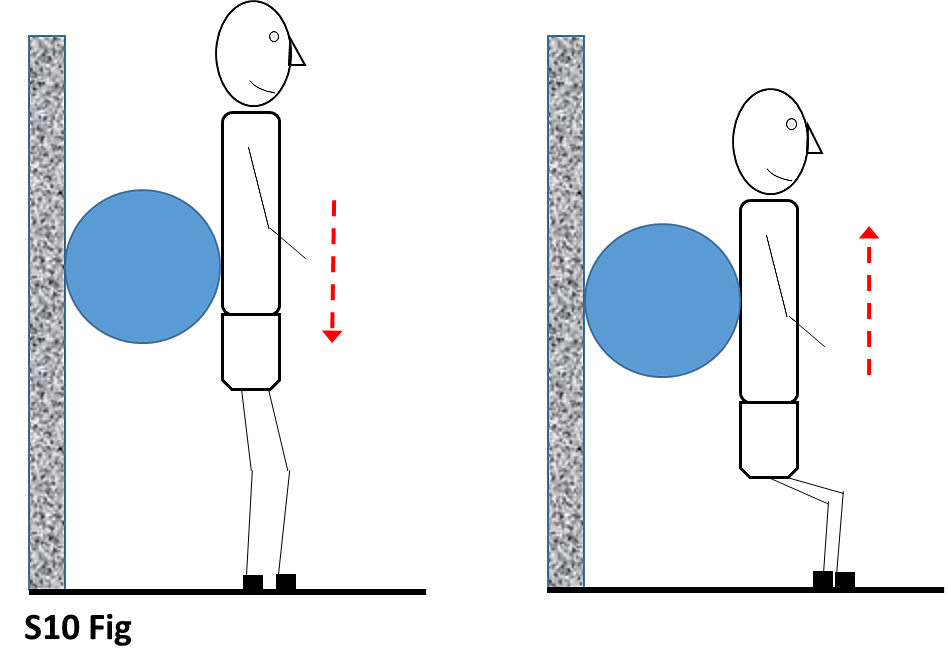


**Exercise 17.** Squats.


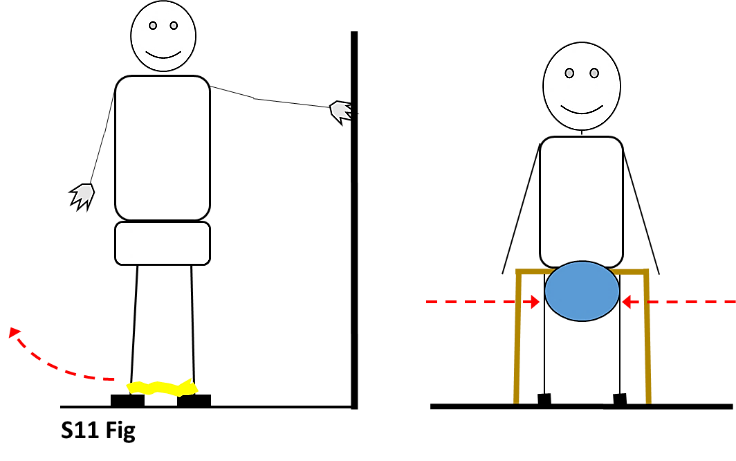


**Exercise 18 & 19.** Hip abduction and adduction.


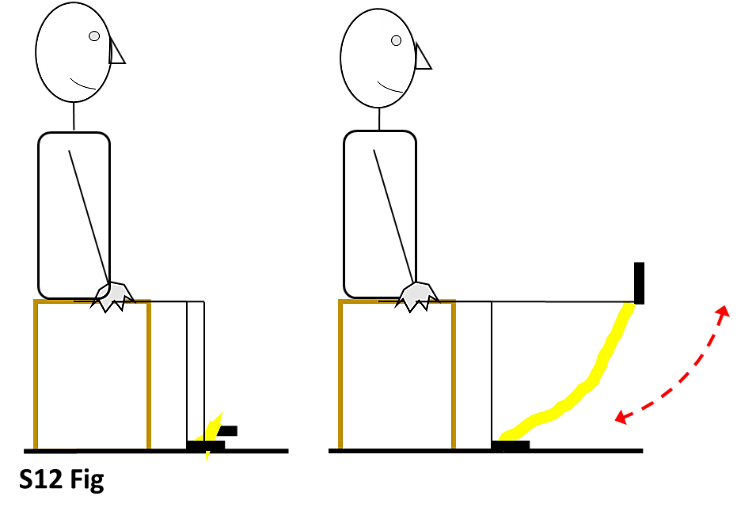


**Exercise 20.** Knee extension.


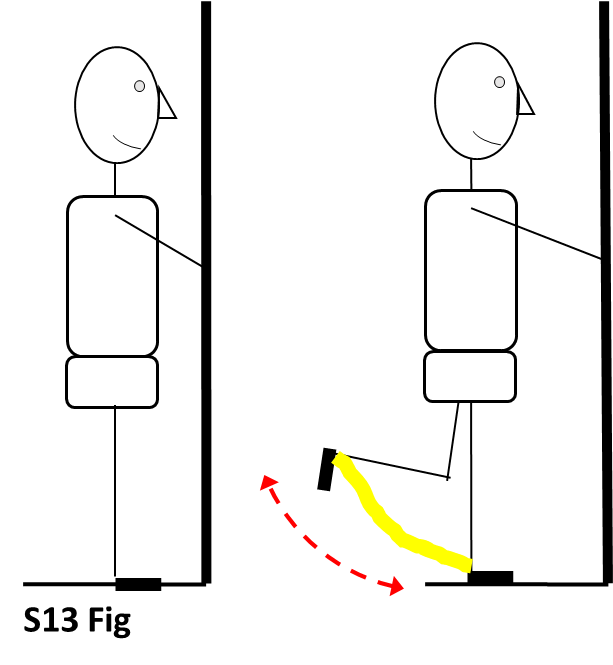


**Exercise 21.** Knee flexion.


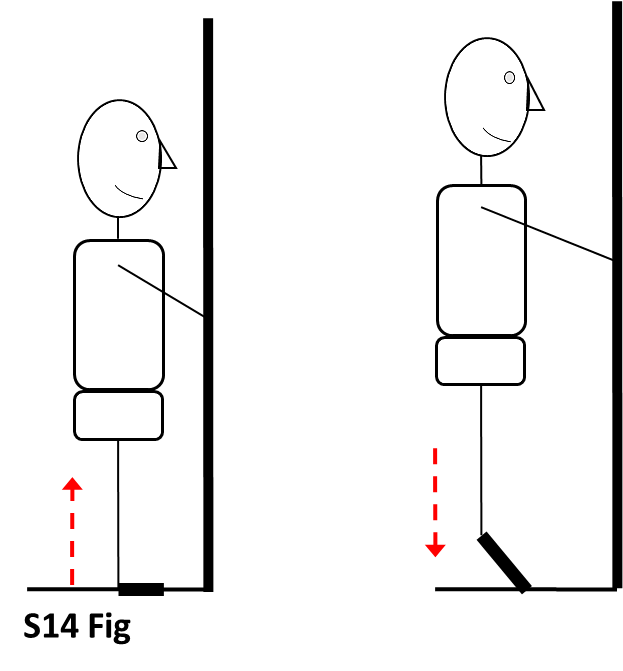


**Exercise 22.** Calf raise.
